# Anti-obesity effect of an isoflavone fatty acid ester on obese mice induced by high fat diet and its potential mechanism

**DOI:** 10.1186/1476-511X-9-49

**Published:** 2010-05-19

**Authors:** Yao Yao, Xiao-Bo Li, Wei Zhao, Yan-Yan Zeng, Hong Shen, Hua Xiang, Hong Xiao

**Affiliations:** 1 Nanjing Brain Hospital affiliated to Nanjing Medical University, Nanjing 210029, PR China; 2 Department of Pharmacy English, China Pharmaceutical University, Nanjing 210009, PR China; 3 Department of Medical Chemistry, China Pharmaceutical University, Nanjing 210009, PR China

## Abstract

**Background:**

The novel compound **1a **is one of the isoflavone fatty acid esters. In order to investigate the anti-obesity effect of compound **1a **and its potential mechanism of influence in adipocyte differentiation, Obese male C57BL/6J mice induced by high-fat diet (HFD) and rat preadipocytes (3T3-L1 cell) were used.

**Methods:**

After 4-week HFD induction, the obese model was made successfully. After treatment with compound **1a**, mice plasma biochemistry parameters were analyzed. In addition, mice hepatic tissue slice was observed. In *in vitro *research, 3T3-L1 cell differentiation by Oil-Red-O staining and adipocyte apoptosis was detected by flow cytometry.

**Results:**

The *in vivo *results implied that compound **1a **significantly decreased the body weight, white adipose tissue weight of obesity mice(p < 0.05), reduced leptin and TG in plasma(p < 0.05), elevated HDL-C in serum(p < 0.05). The *in vitro *results suggested that compound **1a **could significantly suppress the adipocyte viability and lipid accumulation in the differentiation of preadipocyte, and induce apoptosis in both preadipocytes and mature adipocytes(p < 0.05).

**Conclusion:**

Compound **1a **regulates serum lipid profiles, decreases adipose tissue mass and body weight gain by inducing adipocyte apoptosis in high fat diet induced mice. Thus, it may be used to treat obese patients with hypercholesterolemia and hypertriglyceridemia.

## Background

Obesity, which results from a prolonged energy imbalance during which intake exceeds expenditure, is a rapidly growing epidemic in modernized world [1] and is a major risk factor for several serious chronic diseases, such as type 2 diabetes, cardiovascular disease, hypertension, stroke, asthma, and certain forms of cancer [2]. Considerable effort has been devoted to the discovery of anti-obesity drugs worldwide. Though there are dozens of potential targets for anti-obesity, only three agents, sibutramine (Reductil^® ^or Meridia^®^), an appetite suppressant orlistat (Xenical^®^), an inhibitor of fat absorption and rimonaban, a CB1 antagonist, have been introduced into the market during recent years [3]. But several serious adverse effects of sibutramine, orlistat and rimonaban were reported in clinical practice, including gastrointestinal adverse effect and significant unfavorable effects on cardiovascular system [4-6]. The difficulty to lose excessive weight is tightly linked to the complexity and redundancy within lipid systems, which involve an intricate network of peripheral signals and neuronal circuits, constitute obstacles to seek effective potential targets for anti-obesity treatments [7]. In the peripheral organizations, the fat tissue plays an important role in maintaining the energy balance. It is not only an energy storage organ, but also a secretion one. It produces and secretes dozens of factors such as leptin, adiponectin, resistin, visfatin, tumor necrosis factor-α(TNF-α), and interleukin-6 (IL-6), which participate in the energy metabolism of adipose tissue itself and the whole body either in an auto/paracrine or an endocrine fashion [8-11]. Obesity is characterized by an increase in the number and size of adipocytes differentiated from fibloblastic preadipocytes in adipose tissues [12].

Adipocytes play a vital role in regulating adipose mass and obesity, in relation not only to lipid homeostasis and energy balance but also to secreting transcription factors [13]. 3T3-L1 cells have been served as well-established in vitro model to assess adipogenesis and adipocyte differentiation [14]. The programmed differentiation of preadipocytes into adipocytes involves several stages related to obesity [15]. For these reasons, many research efforts have been conducted in 3T3-L1 cells to search for new health benefit foods/agents for obesity or weight control. Potential therapeutic agents that inhibit adipogenesis or increase adipocyte death by apoptosis could be important tools in preventing obesity [16].

Isoflavones are found at high levels in soy plants and structurally similar to estrogens [17]. They are suggested to be a potential alternative to estrogen therapy in the treatment and prevention of menopausal symptoms, osteoporosis, breast cancer and cardiovascular disease. Genistein, daidzein, glycitein, formononetin and biochanin A are considered to be the most important and hence most studied isoflavone phytoestrogens [18,19]. It is also reported that isoflavones have healthful benefits in human obesity and have a positive influence on plasma cholesterol [20].

**Figure 1:**
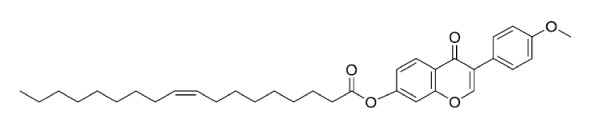
Structural formula of compound 1a.

Here in this paper we defined 4'-methoxy daidzein-7-fatty acid esters as compound **1a **(Fig.1).

We performed research to evaluate the anti- dyslipidemia effect of compound **1a **and its potential mechanism in adipocyte differentiation.

## Materials and methods

### Material

Compound **1a **was provided by China pharmaceutical university. Dulbecco's modified Eagle's medium (DMEM), fetal bovine serum (FBS) were purchased from Gibco BRL of Invitrogen Corporation (Carlsbad, CA). Methylisobutylxanthine, hochest 33342 dye, MTT and Oil-Red-O were obtained from Sigma (St. Louis, MO). Dexamethasone was purchased from Xianju Pharmaceutical Co.Ltd (Zhejiang, China). Insulin was purchased from Wanbang Pharmaceutical Co.Ltd (Xuzhou, China). Leptin ELISA kit was obtained from Boster (Wuhan, China). Anannexin V-fluorescein isothiocyanate (FITC) Apoptosis kit was provided by KeyGene (Nanjing, China). All other reagents were of analytical grade.

### Animals and diets

Fifty male C57BL/6J mice initially weighing 16.03 ± 1.22 g from Nanjing University Animal Center were used. The animals were housed in 12 h light/dark cycle (light on 7a.m.), temperature 22°C, and allowed adlibitum access to diet and water.

#### Animal groups

After one week's acclimatization period, 50 mice were divided randomly into two groups: the normal group (n = 10) were fed with low fat diet (LFD), whereas the experimental group (n = 40) were fed with high-fat diet (HFD).

After 4-week induction, the obese model was made successfully. The 40 obese mice were divided into four groups (n = 10/each group): model group, low dosage group (50 mg·kg^-1 ^compound **1a**), high dosage group (100 mg·kg^-1 ^compound **1a**) and control group (200 mg·kg^-1 ^Inositol Hexanicotinate) respectively.

#### HFD composition

HFD was composed with 90.5% basal diet, 2% cholesterol, 0.5% propacil and 7% grease.

#### Adiminstration modality

Drugs were intragastric administration with suspension in olive oil (0.1 ml/10 g) at 9 A.M. The normal group and model group were administered only olive oil. After 4 weeks, animals were fasted overnight. Next day, blood samples were collected fast from the retroorbital sinus into tubes; serum was separated and stored at -80°C until analysis. Then animals were sacrificed by cervical dislocation and tissues were harvested, weighed, snap frozen in liquid nitrogen and stored at -80°C until use. All animal experiments were approved by the Institutional Animal Care and Use Committees of Nanjing Medical University, and followed National Research Council Guidelines.

### Biochemical analysis

In serum, total cholesterol (TC), high density lipoprotein cholesterol (HDL-C), low density lipoprotein cholesterol (LDL-C) and triglyceride (TG) were measured by Automatic Analyzer 7170A (Hitavhi, Jap.). Serum leptin was detected through leptin ELISA kit (Boster, China).

### Histological analysis

The right lobe of rat liver was fixed in 10% phosphate-buffered formalin for one day and processed in a routine manner to generate 3 μm thick paraffin sections, which were stained with hematoxylin and eosin (HE) and subjected to microscopic examination.

### Cell culture

3T3-L1 preadipocytes were cultured in DMEM supplemented with 10% fetal bovine serum (FBS). After cell reached 80% confluence, the preadipocytes were induced with 1 mg·mL^-1 ^insulin (Wanbang), 1 mmol·L^-1 ^DEX (Xianju) and 0.5 mmol·L^-1 ^MIX (Sigma) (MDI) in DMEM supplemented with 10% fetal bovine serum. After 48 h, the culture medium was replaced with DMEM supplemented with 10% fetal bovine serum and 1 mg·mL^-1 ^insulin, which was switched to DMEM containing 10% fetal bovine serum 2 days later. Cytoplasmic triglyceride droplets were visible under the fluorescence microscope (Carl Ziess Axiovert 40 CFL). For the following experiments, preadipocytes and mature adipocytes were treated for 48 h. All compounds, stock solutions were prepared in DMSO and filter sterilized.

### Cell Viability

All the tests were done in 96-well plates. For non-maturing preadipocyte experiments, cells were seeded (5 × 10^3 ^cells/well) and grown until they reached confluence, and then treated with the compounds or vehicle (DMSO). After the cells were treated with compound **1a **for the 48 h, MTT (Sigma) solution 20 μL (0.5 mg·mL^-1^) was added into the cell culture medium, and the cells were incubated at 37°C and 5% CO_2 _for 4 h. The MTT formazan crystals were then dissolved with 150 μL DMSO and the absorbance in individual wells was determined at 570 nm by a microplate reader (Labsystems Multiskan Ascent). The inhibition of cell growth was evaluated by the MTT method by using triplicate assay. The inhibition ratio was calculated thorough the formula 1.(1)

### Oil-Red-O staining

For Oil-Red-O staining [21], cells were washed gently with phosphate buffered saline (PBS) twice, fixed with 3.7% fresh formaldehyde (Sigma) in PBS for 1 h at room temperature and stained with filtered Oil-Red-O (Sigma) solution (60% isopropanol and 40% water) for at least 1 h. After staining of lipid droplets with red, the Oil-Red-O staining solution was removed and the plates were rinsed with water and dried. Images of the stained lipid droplets were collected on a fluorescence microscope (Carl Ziess Axiovert 40 CFL). The inhibition ratio of lipid production was calculated through formula 1.

### Measurement of the triglyceride content

Cells were seeded in 96-well plates. 3T3-L1 adipocyte monolayer was washed three times with phosphate-buffered saline (PBS) and then fixed for 30 min with 3.7% formaldehyde in PBS. Oil-Red-O (0.5%) in isopropanol was diluted with 2/3 volumes of water, filtered and added to the fixed cell monolayer for 1 h at room temperature. The cell monolayer was then washed with PBS, and the stained triglyceride droplets in the cells were visualized. Finally, the dye retained in the cells was eluted with isopropanol and quantified by measuring the optical absorbance at 492 nm by a microplate reader (Labsystems Multiskan Ascent). The inhibition of lipid accumulation was evaluated by using triplicate assay.

### Apoptosis assays

#### Chromatin Staining with Hoechst 33342

Cells were fixed with 3.7% para-formaldehyde for 30 min, and then stained with 10 μmol·L^-1 ^Hoechst 33342 dye (Sigma) for 30 min in the dark. After staining, cells were observed by fluorescence microscopy (Carl Ziess Axiovert 40 CFL). Cells with bright blue fragmented nuclei showing condensation of chromatin were identified as apoptotic cells.

### Flow cytometry

Phosphatidylserine exposed on the outside of the apoptotic cells was determined by anannexin V-fluorescein isothiocyanate (FITC) Apoptosis kit (KeyGene). In brief, to detect early apoptosis, late apoptosis, and necrosis induced by compound **1a**, we plated the 3T3-L1 adipocytes at a density of 1 × 10^6 ^cells/dish in a 60-mm dish and exposed them for 24 h at 37°C. Cells were harvested by centrifugation and washed with ice-cold PBS twice; the cells were then stained with 20 μL of annexin V-FITC and 20 μL of PI at room temperature for 15 min in the dark. Annexin V-FITC and PI emissions were detected in the FL1 and FL2 channels of a FACScan flow cytometer (BD FACSC anto™), using emission filters of 525 nm and 575 nm. The percentages of distribution of normal cells and cells in early apoptosis, late apoptosis and necrosis were calculated by CellQuest software (BD Biosciences).

### Statistical Analysis

Data are presented as the mean ± SD. Data were analyzed by one-way ANOVA using Statistical Package for Social Science (SPSS, Chicago, IL). Differences were considered to be significant at p < 0.05.

## Results

### Body mass (BM) and fat mass

**Figure 2:**
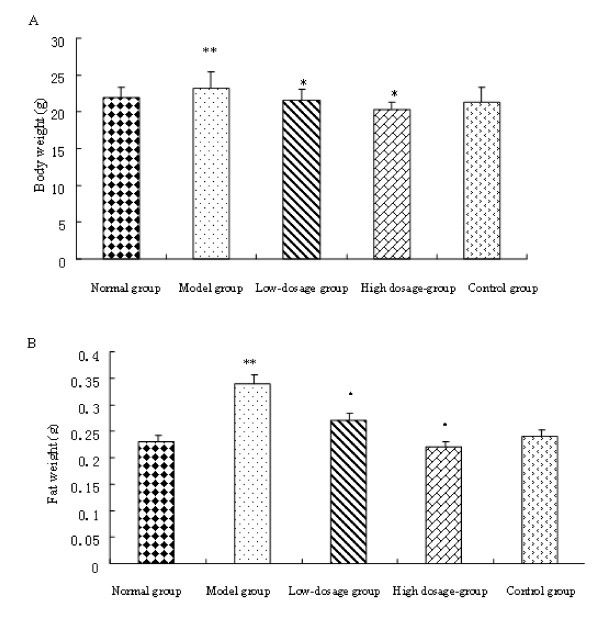
**Effects of compound **1a **on C57BL/6J mice**. **A**: Effects of compound **1a **on the weight of the mice after treatment. **B**: Effects of compound **1a **on the weight of the adipose tissue after treatment. Normal group: low fat diet; Model group: high fat diet; Low dosage group: high fat diet+50 mg·kg^-1 ^compound **1a**; High dosage group: high fat diet+100 mg·kg^-1 ^compound **1a**; Control group: high fat diet+200 mg·kg^-1 ^Inositol Hexanicotinate. All values are mean ± SD (n = 10), * presents P < 0.05 compared with model group. ** presents P < 0.05 compared with normal group

After 4 weeks' induction, mice fed with HFD had 5.3% higher body weights compared with normal group fed with LFD (p < 0.05). The obese model was made successfully. Four-week treatment later, low dosage group and high dosage group had 6.86% and 12.33% lower body weights (p < 0.05) than model group respectively (Fig.2A). No obvious difference was found between normal group and high dosage group. As compound **1a **dosage doubled, the weight loss compared with model group increased. The weight loss displayed dosage dependence.

White adipose tissue mass was also significantly increased by 148% in model group compared with normal group (p < 0.05) (Fig.2B). In response to compound **1a **at high dosage, the adipose tissue weights in high dosage group were significantly decreased by 35.29% compared with the model group (p < 0.05). The result inferred that compound **1a **inhibited HFD-induced body weight gain and adipose tissue mass in mice effectively.

### Changes in lipid profiles

**Figure 3:**
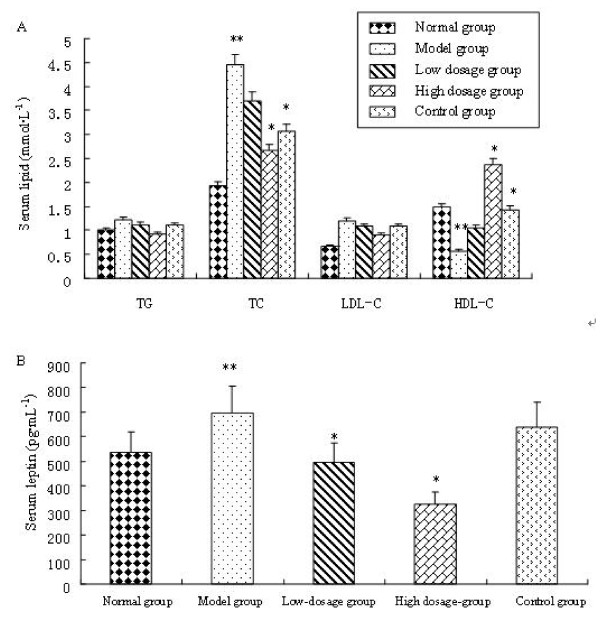
**Effects of compound **1a **on serum lipids and leptin. A**: Changes in TG, TC, LDL-C and HDL-C by compound **1a **in high-fat diet fed obese mice. Compound **1a**-treated mice had significantly lower serum concentration of triglycerides and higher HDL-C compared with high-fat diet alone. **B**: Effects of compound **1a **on serum leptin. Serum leptin concentration was reduced by compound **1a **in a concentration dependent manner. Normal group: low fat diet; Model group: high fat diet; Low dosage group: high fat diet+50 mg·kg^-1 ^compound **1a**; High dosage group: high fat diet+100 mg·kg^-1 ^compound **1a**; Control group: high fat diet+200 mg·kg^-1 ^Inositol Hexanicotinate. All values are mean ± SD (n = 10), * presents P < 0.05 compared with model group. ** presents P < 0.05 compared with normal group.

To determine whether compound **1a **has beneficial effects on the circulating lipid profiles, we tested its effects on serum triglyceride and cholesterol levels. Compared with normal group, serum levels of triglycerides and total cholesterol in model group were increased by 21.32% (p < 0.05) and 164% (p < 0.05) respectively (Fig.3A). This result suggested that the high lipid model was made successfully. After treatment, compound **1a **(high dosage) caused significant reductions in serum triglyceride and total cholesterol levels by 28% (p < 0.05) and 46% (p < 0.05) respectively, compared with model group. It was worthy noted that serum HDL-C in high dosage group was much higher than that in other groups (p < 0.05). Consequently, we believed that compound **1a **suppressed lipid disorders through increasing HDL-C and depressing TG, thereby preventing hypertriglyceridemia and hypercholesterolemia.

### Changes in serum leptin

The serum leptin level in model group was much higher than that in normal group (p < 0.05). The result implied that obesity mice accompanied with leptin resistance. Compound **1a **(high dosage) caused significant reduction in serum leptin by 52.21% (p < 0.05) compared with model group and reduced serum leptin in a dose- dependent manner (Fig. 3B).

The results suggested that compound **1a **could improve leptin resistance induced by obesity.

### Hepatic lipid accumulation

The hepatic accumulation of lipid was observed in mice hepatic tissue slice by light microscopy. Mice in model group showed considerable hepatic lipid accumulation compared with that in normal group.

**Figure 4:**
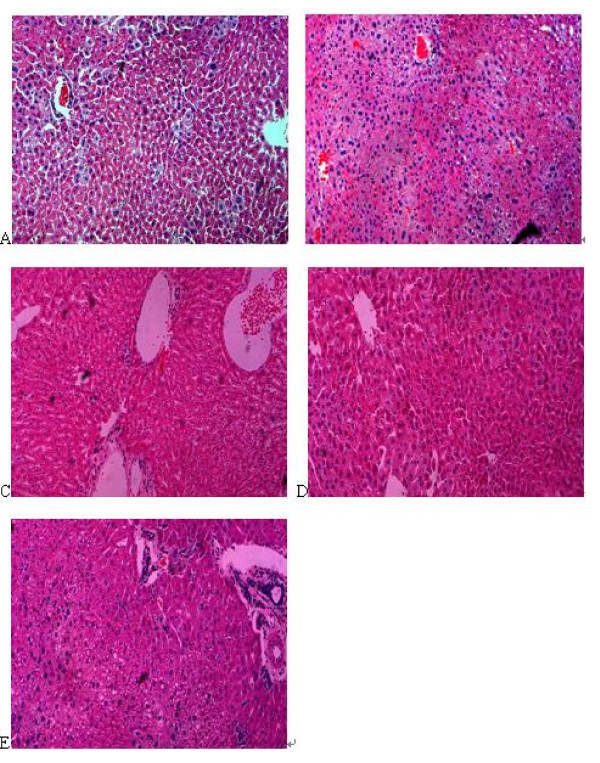
**Inhibition of hepatic lipid accumulation by compound **1a **in high-fat diet-fed obese mice. **Histological analyses of hepatic lipid accumulation. Representative HE-stained liver sections are shown (original magnification 200×). **A **presents the normal hepatocyte appearance. High fat diet induced hepatocytes necrosis was shown in **B**. After the treatment of compound **1a**, necrosis hepatocytes are reduced, especially in **D**. Small lipid drops were seen **C**. There were obvious lipid drops in **E**. **A**: low fat diet (normal group); **B**: high fat diet (model group); **C**: high fat diet+50 mg·kg^-1 ^compound **1a **(low dosage group); **D**: high fat diet+100 mg·kg^-1 ^compound **1a **(high dosage group); **E**: high fat diet+200 mg·kg^-1 ^Inositol Hexanicotinate (control group).

The mice hepar in high dosage group showed considerably lower hepatic lipid accumulation than in model group. As shown in Fig.4, the triglyceride droplets appeared in mice hepar were almost completely disappeared by compound **1a **treatment. Thus, the result suggested that compound **1a **could increase fat catabolism in the liver and inhibited hepatic lipid accumulation.

### Proliferation and differentiation of 3T3-L1

**Figure 5:**
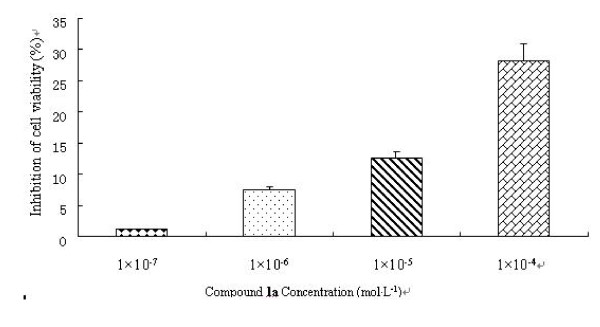
**Effect of compound **1a **on the inhibition of cell viability in 3T3-L1 preadipocytes. **After the influence reached 80%, 3T3-L1 preadipocytes were incubated with compound **1a**, at the indicated concentration (1 × 10^-7^, 1 × 10^-6^, 1 × 10^-5^, 1 × 10^-4^mol L^-1^) for 48 h; growth rate was assessed by MTT assay. The inhibition ratio was calculated by formula 1. All values are mean ± SD (n = 3)

**Figure 6:**
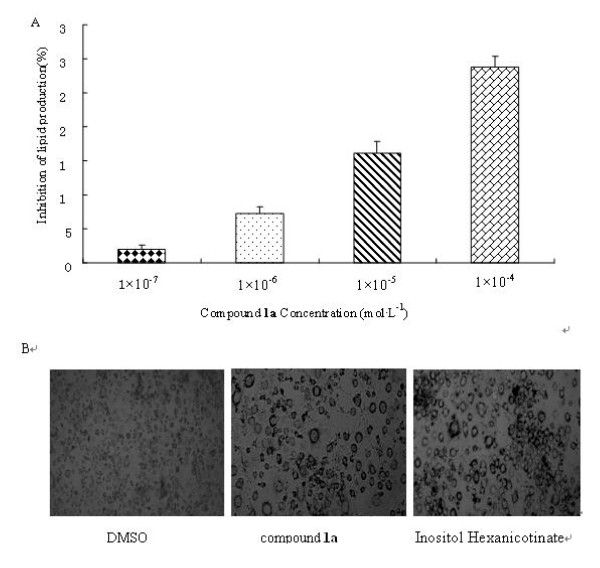
**Effect of compound **1a **on lipid adipogenesis. A**: Effect of compound **1a **on lipid accumulation in 3T3-L1 adipocytes. At two days after confluence, 3T3-L1 cells were differentiated by MDI. At day 8 compound **1a **was given to the cells at series of concentrations. Ten days after differentiation, we measured triglyceride content of 3T3-L1 cells treated with compound **1a **by Oil-Red-O staining. The inhibition ratio was calculated by formula 1. **B**: Effect of vehicle, compound **1a**, Inositol Nicotinate on the lipid accumulation by Oil-Red-O staining. Images of the stained lipid droplets were collected on a fluorescence microscope. Lipid accumulation was significantly decreased treated by compound **1a**. All values are mean ± SD (n = 3).

To determine whether compound **1a **inhibited the cell viability of 3T3-L1 preadipocytes, cells were treated with four various doses of compound **1a **and Inositol Hexanicotinate. Cell viability was estimated by the MTT assay and compound **1a **treatment inhibited cell growth in a dose-dependent manner (Fig.5). When it reached 0.1 mmol·L^-1^, the viability was suppressed by 28.31%. To examine the effect of compound **1a **on adipogenesis, 3T3-L1 preadipocytes were treated with adipogenic hormone mixture in the absence or presence of compound **1a**. During 3T3-L1 cell differentiation, the number of larger lipid droplets in cells decreased. After 10 days, triglyceride content of 3T3-L1 cells treated with compound **1a **was measured. It exhibited a significant dose-dependent decrease in the intracellular accumulation of triacylglycerol under Oil-Red-O staining (Fig.6). However, there was no significant difference between Inositol Hexanicotinate group and the control group.

### Apoptosis assays

An increase in apoptotic nuclei in both preadipocytes and mature adipocytes treated with 0.1 mmol·L^-1 ^compound **1a **were evident from photos of Hoechst-33342 staining (data not show). We suggested that apoptosis was induced by the compound **1a **both in preadipocytes and in mature adipocytes.

**Figure 7:**
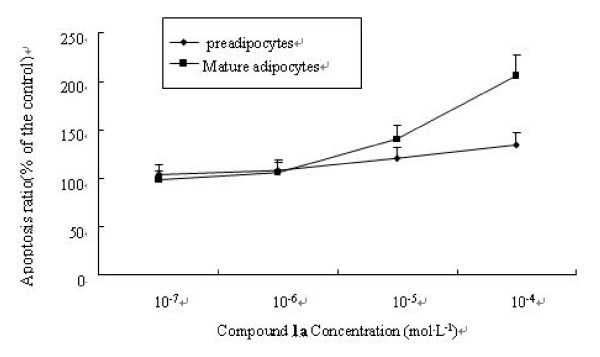
**Apoptosis ratios (apoptosis cell number in treatment group/apoptosis cell number in blank group) after 24 h were shown in this picture. **With the concentration increase, the apoptosis ratio was raised both in preadipocytes and mature adipocytes. The phenomenon was more notable in mature adipocytes induced by 1 × 10^-4 ^mol·L^-1 ^compound **1a**, whose apoptosis ratio reached almost 206% compared with DMSO group.

To quantify the modes of cell death induced by compound **1a**, 3T3-L1 preadipocytes and adipocytes were treated with series concentration of compound **1a**(1 × 10^-4^, 1 × 10^-5^, 1 × 10^-6^, 1 × 10^-7^mol·L^-1^) for 24 h, were subjected to simultaneous staining with annexin V-FITC and PI, and were analyzed by FACScan flow cytometry. Annexin V-FITC/PI double-staining analysis demonstrated that the number of normal cells decreased in a dose-dependent manner exposed to compound **1a. **The apoptotic cells, including early apoptosis and late apoptosis, were increased in a dose-dependent manner. When the compound **1a **concentration elevated to 0.1 mmol·L^-1^, the percentage of cells in G3 decreased from 52.2% to 35.9% (preadipocytes) and from 86.1% to 72% (adipocytes), respectively; cells in G2 and G4, including early and late apoptosis, increased from 47.8% to 64.1% (preadipocytes) and from 13.3% to 27.4%(adipocytes), respectively (Table.1, Fig.7).

**Table 1:** Preadipocytes(table.1-1) and mature adipocytes(table.1-2) were incubated in vitro without or with 1 × 10^-7^, 1 × 10^-^^6^, 1 × 10^-^^5^, 1 × 10^-4 ^mol·L^-1 ^of compound **1a **for 24 h and analyzed by Annexin V/PI cell viability assay.

1-1					
**%Parent**	**blank**	1*10^-7^	1*10^-6^	1*10^-5^	1*10^-4^

Q1	1.7	2.5	1.8	2.8	1.5

Q2	39.0	38.5	39.2	41.6	43.2

Q3	50.5	47.6	46.7	39.5	34.4

Q4	8.8	11.2	12.4	16.0	20.9

**1-2**					

Q1	0.5	0.2	0.5	0.1	0.5

Q2	1.8	1.3	1.5	1.4	2.7

Q3	86.1	85.8	85.4	81.2	72

Q4	11.5	12.8	12.6	17.3	24.7

Apoptotic (Q4+Q2) cells were identified and expressed as a percentage of total dead cells.

The result demonstrated that compound **1a **could induce apoptosis of both preadipocytes and mature adipocytes.

## Discussion

Obesity is the result of an energy imbalance caused by an increased ratio of caloric intake to energy expenditure. In conjunction with obesity, related metabolic disorders such as dyslipidemia, atherosclerosis, and type 2 diabetes have become global health problems. As well as diet and exercise, some drugs are useful for weight reduction. To date, only sibutramine and orlistat have been approved for use, though both have poor outcomes and are not so well tolerated [22]. Rimonabant is a selective CB1 receptor blocker which has been proven effective in achieving substantial weight loss and in reducing triglyceride levels and glycohemoglobin. It has also been shown to increase HDL cholesterol levels in obese patients and specifically in patients with diabetes [23-25]. However, it was reported that sibutramine, orlistat and Rimonabant had several serious adverse effects in clinic, including gastrointestinal adverse effect and significant unfavorable effects on cardiovascular system. As a result, much safer therapeutic is necessary. In addition, there has been a large increase in the use of complementary treatments such as herbal remedies in the treatment of these diseases over the past decade [26].

We observed that mice fed with HFD supplemented with compound **1a **for 4 weeks had lower levels of serum triglycerides, LDL-C and higher levels of HDL-C compared with mice fed with HFD alone, which indicated that compound **1a **efficiently regulated triglyceride and cholesterol metabolism in obese mice. Thus, compound **1a **may be beneficial for treating patients with hypercholesterolemia and hypertriglyceridemia.

Lipids are transported in the body by a sophisticated lipoprotein transport system. Lipoproteins are divided into 5 main classes according to their density: chylomicrons, VLDL-C, IDL-C, LDL-C and HDL-C [27]. LDL-C is a risk factor for CAD. The antagonist of LDL-C is HDL-C. It collects cholesterol from body tissues and returns it to the liver. As a result, HDL-C cholesterol levels are inversely related to this risk [28]. Compound **1a **was determined to effectively in elevating HDL-C. Thus, we tentatively put forward that compound **1a **was effective in reversing coronary artery disease.

In parallel with its effect on serum TG, mice fed with HFD supplemented with compound **1a **had significantly lower adipose tissue mass and body weight gain than that fed with HFD alone. This correlated with reports showed that the lipids in adipose tissue were largely derived from circulating triglycerides, especially during HFD feeding [29,30] and that the reductions in serum TG also led to decreased adipose tissue mass [31-33].

Increased lipid accumulation in hyperlipidemic rats and mice may be due to increased levels of triglyceride depots in the liver [33]. Histological analysis in our research showed that HFD-fed mice had considerable hepatic lipid accumulation. After 4-week treatment with compound **1a**, hepatic lipid accumulation disappeared (high dosage group). Long term hepatic lipid accumulation would cause fatty liver. Thus the result suggested that compound **1a **could recover fatty liver induced by lipid accumulation.

Leptin, a 167-amino acid protein, is synthesized and secreted mainly by adipose tissue [34,35]. Adipose tissue produces the hormone leptin in approximate proportion to fat stores. Circulating leptin communicates the level of energy reserves in the periphery to the central nervous system (CNS) in order to suppress food intake and permit energy expenditure [34,36-38]. Peripheral administration of exogenous leptin results in a specific reduction in body fat mass with no change in lean mass in experimental animals [39] or in obese humans on a weight-reducing diet [40], but elevated concentrations of endogenous leptin do not appear to be capable of preventing, or reversing, the accumulation of adipose tissue during, or after, the development of obesity [41]. Several authors have reported that aging [42,43] or the consumption of a HFD [44-46] results in the development of leptin resistance in rodents, measured as a failure of leptin either to inhibit food intake or to induce weight loss. Increased circulating leptin, a marker of leptin resistance, is common in obesity and independently associates with insulin resistance [47] and CVD [48-51] in humans. In our study, Serum leptin in model group was raised during the induction of HFD to 694.76 pg·mL^-1^. After the treatment of 100 mg·kg^-1 ^compound **1a**, the serum leptin was reduced to 52.21% compared with model group. It suggested that compound **1a **was effective to leptin resistance induced by HFD in mice.

The possible mechanisms for the treatment of obesity are as follows: balance energy intake and expenditure, reduce preadipocyte differentiation, decrease lipogenesis, increase lipolysis and induce adipocyte apoptosis [52]. Adipocytes play an important role in lipid homeostasis and energy balance by relating to triglyceride storage and free fatty acids release. Adipocyte differentiation and the amount of fat accumulation are associated with the occurrence and development of obesity [53]. Moreover, adipocyte produces and secretes dozens of factors such as leptin, adiponectin, resistin, visfatin, TNF-α, and IL-6, which participate in energy metabolism of adipose tissue itself and the whole body either in an auto/paracrine or an endocrine fashion [54]. For these reasons, anti-obesity works have been conducted in 3T3-L1 adipocytes.

Anti-obesity effect of compound **1a **in the 3T3-L1 cell model was measured in our study. Any reduction in adipocyte number can result from preadipocyte or adipocyte apoptosis, as well as adipocyte dedifferentiation [55]. In this study we investigated the effects of compound **1a **on adipocyte viability, apoptosis and lipid accumulation during adipogenesis in 3T3-L1 cells. We found that compound **1a **inhibited cell viability and enhanced apoptosis in both pre- and mature adipocytes. Correspondingly, the lipid accumulation was suppressed during the differentiation process. As to *in vivo *experiment, the adipose was depauperated, which was coincident with the weight and adipose tissue loss.

Lipolysis is a property of mature, differentiated adipocytes. Adipocyte maturation requires an elaborate sequence of events associated with specific patterns of transcription factors at different phases of differentiation [56]. Mature adipocytes have massive TG storage compared with any other tissue, despite having a well-developed lipolytic capacity. Conversely, a decrease of adipocyte differentiation, which by definition is associated with reduction of the lipid droplet volume, will transiently be accompanied by a net increase in lipolysis, and this is one potential mechanism for increasing lipolysis. As an important component in the network of lipid energy metabolism, adipocyte lipolysis is positioned to affect the fat content of liver, muscle, heart and kidney in obesity.

In conclusion, the results of the in vivo and in vitro study show that compound **1a **regulates serum lipid profiles, decreases adipose tissue mass and body weight gain by inducing adipocyte apoptosis. The compound **1a **may be used to treat obese patients with hypercholesterolemia and hypertriglyceridemia. The adipocyte apoptosis mechanism induced by compound **1a **will be further researched.

## Abbreviations

CAD: Coronary Artery Disease; CNS: Central nervous system; CVD: Cardiovascular disease; DEX: Dexamethasone; DMEM: Dulbecco's modified Eagle's medium; FBS: Fetal bovine serum; HFD: High fat diet; HDL-C: High density lipoprotein cholesterol; HE: Hematoxylin and eosin; IBMX: 3-Isobutyl-1-methylxanthine; IL-6: Interleukin-6; LDL-C: Low density lipoprotein cholesterol; LFD: Low fat diet; MTT: 3-(4, 5-Dimethylthiazol-2-yl)-2, 5-diphenyltetrazolium bromide; PBS: Phosphate buffered saline; TC: Total cholesterol; TG: Triglyceride; TNF-α: Tumor necrosis factor-α.

## Competing interests

The authors declare that they have no competing interests.

## Authors' contributions

YY carried out both in vivo and in vitro studies and drafted the manuscript. LXB participated in vivo study and helped to draft the manuscript. ZW carried out the synthesis of compound 1a. ZYY cultured the cells. SH carried out the determination of plasma leptin and data analysis. XH and XH conceived, design the research. All authors have read and approved the final manuscript.
